# Impact of community-based adherence support on treatment outcomes for tuberculosis, leprosy and HIV/AIDS-infected individuals in post-Ebola Liberia

**DOI:** 10.1080/16549716.2018.1522150

**Published:** 2018-10-01

**Authors:** Julia H. Rogers, Lassana Jabateh, Jason Beste, Bradley H. Wagenaar, Ryan McBain, Daniel Palazuelos, Eugene Wickett, Catherine Oswald, Harriet G. Napier, Julia Toomey-Garbo

**Affiliations:** a Partners In Health Liberia, Monrovia; b Brigham and Women’s Hospital, Boston, MA; c Harvard Medical School, Boston, MA; d Department of Global Health, University of Washington, Seattle, WA; e Health Alliance International, Seattle, WA; f Department of International Health, Johns Hopkins University, Baltimore, MD; g Liberian Ministry of Health, Monrovia

**Keywords:** Community health worker (CHW), socioeconomic assistance, health systems strengthening, post-emergency setting, observational study

## Abstract

**Background**: Partners In Health (PIH) committed to improving health care delivery in Maryland County, Liberia following the Ebola epidemic by employing 71 community health workers (CHWs) to provide treatment support to tuberculosis (TB), HIV and leprosy patients. PIH simultaneously deployed a socioeconomic assistance program with three core components: transportation reimbursement to clinics; food support; and additional social assistance in select cases.

**Objective**: This study aimed to evaluate how a CHW program for community treatment support and addressing socioeconomic barriers to care can impact patient outcomes in a post-conflict and post-epidemic context.

**Methods**: Retrospective observational study utilizing registry data from 513 TB, 447 HIV and 75 leprosy patients at three health facilities in Maryland County, Liberia. Treatment coverage and clinical outcomes for patient cohorts enrolled in the pre-intervention period (January 2015 to June 2015) and the post-intervention period (July 2015 to July 2017) are compared using logistic regression analyses.

**Results**: TB treatment coverage increased from 7.7% pre-intervention to 43.2% (p < 0.001) post-intervention and lost to follow-up (LTFU) rates decreased from 9.5% to 2.1% (p = 0.003). ART treatment coverage increased 3.8 percentage points (p = 0.03), with patient retention improving 63.9% to 86.1% (p < 0.001); a 6.0 percentage point decrease in HIV LTFU was also observed (p = 0.21). Despite an 84.3% treatment success rate observed for leprosy patients, pre-intervention data was largely unavailable and statistical significance could not be reached for any treatment outcomes pre-post intervention.

**Conclusions**: The PIH approach to CHW community treatment support in Liberia demonstrates how, with the right inputs, excellent clinical outcomes are possible even in post-conflict and post-epidemic contexts. Care should be taken to position and support CHWs so that they have the opportunity to succeed, including full integration and recognition within the system, and the addition of clinical system improvements and social supports that are too often dismissed as unsustainable.

## Background

Liberia’s health system has suffered three major upheavals in the last three decades: two civil wars that resulted in an estimated 150,000–300,000 deaths and the internal displacement of 220,000 persons [1-2], and an Ebola virus disease (EVD) epidemic that caused 4,806 deaths [3]. Over 80 of these deaths were health professionals, which represented 8% of Liberia’s health workforce [4]. Prior to the 2014 EVD outbreak, Liberia’s Human Development Index was just 0.39, ranking 174 out of 187 countries [5], and the national representation of health workers included just one health worker for every 100,000 people [6].

This loss of human resources – and distrust in the health care system during and immediately following the EVD outbreak – had major effects on health outcomes. A series of surveys conducted by the United Nations found a substantial decline in the number of persons seeking health care in Liberia due to fear of nosocomial EVD transmission, leading to increased maternal mortality, low immunization rates, and a significant burden of infectious disease among children under five [7]. Another study found that health systems outputs for 10 key primary health care indicators decreased between 35% and 67% during the EVD outbreak, as well as a 49.2% increase in malaria cases when comparing pre-EVD with post-EVD data [8]. The EVD outbreak also disproportionately affected the more remote counties of Liberia due to inequality in the distribution of health care resources: over 60% of health workers work in and around Monrovia, where just 30% of the Liberian population reside [7].

Predictably, treatment for chronic infectious diseases requiring long-term or repeated medical care was greatly affected, particularly for tuberculosis (TB) and HIV. One study found that the number of antiretroviral therapy (ART) patient visits per week at one clinic in Monrovia abruptly decreased by 59%, and progressively decreased by 3% per week at a second clinic [9]. In 2015, Liberia was declared by the World Health Organization (WHO) as one of nine high TB/HIV burden countries in which less than 50% of TB patients living with HIV had access to ART [10]. Reliable information on the effect of the EVD outbreak on mortality rates of HIV and TB are limited; however, it has been estimated through modeling that the outbreak resulted in a 13% increase in HIV/AIDS mortality and a 59% increase in TB mortality nationwide [11].

Existing research suggests that community health workers (CHWs) can be effective in mitigating risks of transmission during infectious disease outbreaks and supporting community-based adherence and retention in care for TB and HIV patients. A systematic review comparing treatment outcomes of CHW-administered Directly Observed Treatment Short-course (DOTS) versus facility-administered DOTS for TB patients found that community-based methods outperformed facility DOTS treatment success rates, while another reported clear evidence of CHWs’ contribution to HIV services delivery and outcomes in sub-Saharan Africa [12–15]. Despite these studies, insufficient evidence has been published offering effective solutions as to how CHWs may be integrated into the primary health care system to bridge substantial rural health delivery gaps and improve care and management of chronic infectious diseases, especially in post-emergency settings [13,16]. In countries with comparable HIV, TB and leprosy epidemiology with similar histories of civil war and epidemics to Liberia (such as the Democratic Republic of the Congo and Sierra Leone), published results on the impact of lay person cadres providing treatment support for these diseases are limited [17–22].

Employing lessons learned from similar work in other fragile and under-resourced health systems, we worked to strengthen the health system at central clinical facilities and deployed a cadre of CHWs to identify patients and support their treatment, alongside the inclusion of a variety of social and economic inputs to address vulnerability and augment the agency necessary to adhere to chronic care regimens. The program’s comprehensive strategy – conceived in parallel with Liberia’s national community health policy revision – mandates investment in an integrated community health structure as a prerequisite to achieving functionality of the overall health system.

The aim of this manuscript is to extend previous research that evaluates how CHW programs can contribute to health systems strengthening efforts in a post-conflict or epidemic context. Our findings and recommendations may serve as a practical guide for program implementers in adopting an integrated community treatment support model that addresses the target population’s socioeconomic vulnerabilities within Ministry of Health (MOH) guidelines and national strategic plans.

## Methods

### Study setting

Partners In Health (PIH), a non-governmental organization whose mission is to provide the highest quality care possible to the sick and impoverished, was invited by the Liberian government in October 2014 to address the root causes of EVD by strengthening the health system in Maryland County, despite the county only reporting seven confirmed or probable cases of the disease. Situated in Southeastern Liberia, Maryland is home to an estimated 150,731 residents, 84% of whom suffer from absolute poverty and 72% from food poverty – the highest rates when compared to the rest of the country [23]. At the onset of the intervention, there were 21 public health facilities in the county and three privately-operated health facilities.

### Intervention description

PIH and the Maryland County Health Team formalized the community health program and initial recruitment of a CHW cohort in May 2015. Literacy levels of the CHWs were not evaluated during the recruitment process. CHWs work out of three health facilities, two public and one private, that were the sole providers of HIV, TB, and leprosy treatments in the county at the time of the program’s implementation. Leprosy was included as a targeted disease due to the integration of leprosy and TB control programs in Liberia.

Maryland County is divided into six health districts. The public facilities supported by PIH – Pleebo Health Center and J.J. Dossen Hospital – are located in two contiguous districts and receive the highest volume of patients in the county: their combined catchment populations of 70,513 account for 43.9% of the county population. The private facility, St. Francis Health Clinic, is located in the same district and catchment area as Pleebo Health Center but was included as a facility to receive support through our CHW program due to it being the only facility in the district providing ART at the time of the intervention’s initiation. Private and public facilities in the county utilize the same patient registers and reporting forms submitted to the National AIDS and STI Control Program (NACP) and National Leprosy and Tuberculosis Control Program (NLTCP), and all TB, HIV and leprosy treatments are free of charge.

We designed the program to meet five main objectives: (1) improve clinical outcomes by extending HIV, TB and leprosy services (clinical and social support) through a CHW network integrated into, and recognized within, the health system; (2) build a strong bi-directional referral pathway and serve as a bridge between the community and primary health care system; (3) empower CHWs through training, supervision (peer and clinical), adequate remuneration, and integration; (4) address delays in decision to seek care, delays in reaching care, and delays in receiving care; and (5) increase the rate of patient enrollment in care through community-based case finding, and decrease the rates of lost to follow-up (LTFU) patients.

By April 2016, we completed the full recruitment and training of 71 CHWs and seven Peer Supervisors in Maryland County. All active CHWs and Peer Supervisors live within the catchment areas of the three PIH-supported facilities. As of June 2017, the mean number of patient home visits that a CHW conducts each month is 70, and the mean number of patients assigned to each CHW is eight.

**Figure 1.**
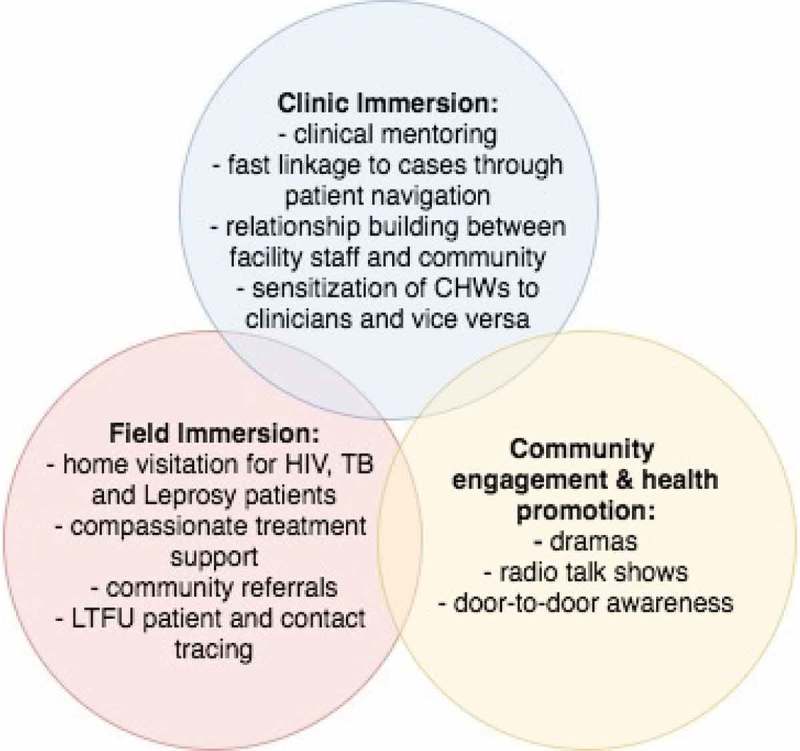
The three primary components of CHW activities.

**Figure 2.**
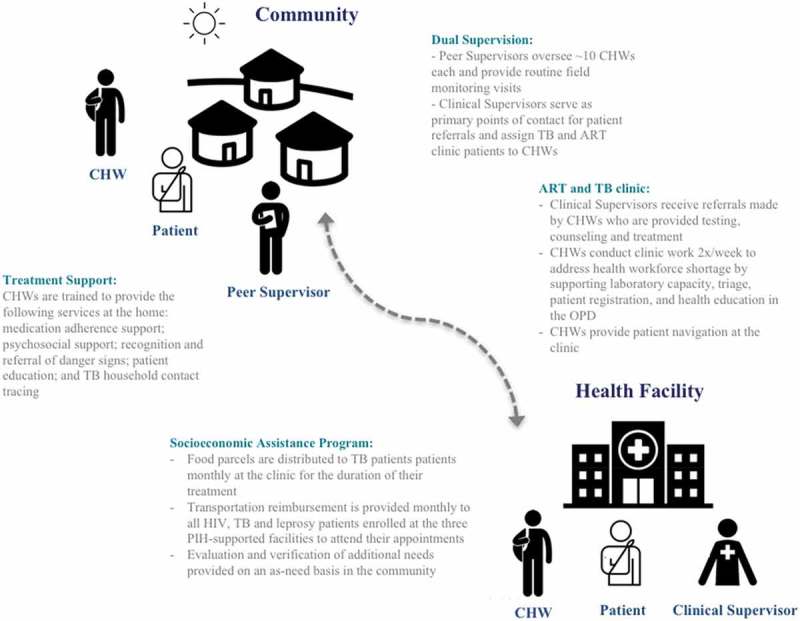
Integrated community and facility continuum of care for TB, leprosy and HIV patients in Maryland County.

The application of an integrated approach to patient care includes addressing the social determinants of health that act as barriers to treatment adherence. For this reason, we devised a socioeconomic assistance program with three core components: (1) transportation reimbursement; (2) food support packages; and (3) additional social assistance. All HIV, TB and leprosy patients are eligible for components one and three. Only TB patients receive monthly food packages – containing sardines, beans, rice, and vegetable oil – given the strong bidirectional association between undernutrition and TB. The PIH-employed Social Protection Officer manages the program and, alongside the CHWs, is responsible for assessing the socioeconomic needs of various patients and tracking the progress of the program’s beneficiaries. Additional social assistance provided is also determined by the Social Protection Officer, most often in the form of cash transfers for safe housing, school fees, or auxiliary nutritional packages for patients lacking familial support. Figures 1 and 2 list key components of the CHW program as it relates to the continuum of care for HIV, TB and leprosy patients. This includes: a dual supervision structure, clinic immersion for the CHWs, and the socioeconomic assistance program.

### Data sources and measures

Data was collected on a monthly basis from three sources: the CHW, the Peer Supervisor, and the Clinical Supervisor of the ART clinic or chest clinic. PIH recorded data pre- and post-intervention via this method. All reporting tools were paper-based and collected and analyzed by data clerks in PIH’s Monitoring & Evaluation (M&E) department. Clinical Supervisors submitted reports to the M&E data clerks that mirrored those submitted quarterly to both the NLTCP and the NACP, with several additional indicators intended to assess impact of the program’s enhancements. All patient outcome information is extracted from a column found in the NLTCP and NACP clinic registers. These data are integrated with the national MOH’s District Health Information System (DHIS-2) and monthly outputs are reported to the national level for program monitoring and evaluation. All reporting tools and data collection methods used for HIV, TB, and leprosy treatment outcomes at both the private and public facilities follow standardized guidelines by NACP and NLTCP, with all data being aggregated at the county-level. All pre-EVD outcomes and enrollment data reported in Table 1 are from the national DHIS-2.

**Table 1.** Treatment coverage and outcomes for HIV, TB, and leprosy patient cohorts enrolled pre-EVD (measured at December 2013), pre-intervention (measured at June 2015) and post-intervention (measured at July 2017) in all PIH-supported facilities across Maryland County, Liberia.

	Pre-EVD (Jan 2013–Dec 2013); n (%)	Pre-intervention (Jan 2015–Jun 2015); n (%)	Post-intervention (Jul 2015–Jul 2017); n (%)	Chi-square statistic; p-value
HIV				
*Total new enrollments in period range*	180	36	411	
*Still enrolled*	60 (33.3)	23 (63.9)	354 (86.1)	χ^2^ = 12.40; p < 0.001
*% treatment coverage*	N/A	30.6	34.4	χ^2^ = 4.87; p = 0.03
*% treatment coverage in PIH-supported districts*	N/A	48.0	53.9	χ^2^ = 6.96; p = 0.008
*Transfer Out*	24 (13.3)	6 (16.7)	8 (1.9)	χ^2^ = 23.64; p < 0.001*
*LTFU*	65 (36.1)	5 (13.9)	32 (7.8)	χ^2^ = 1.62; p = 0.21*
*Died*	31 (17.2)	2 (5.6)	17 (4.1)	χ^2^ = 0.16; p = 0.66*
	Pre-EVD (Jan 2013–Dec 2013); n (%)	Pre-intervention (Jan 2015–Jun 2015); n (%)	Post-intervention (Jul 2015–Sep 2016); n (%)	Chi-square statistic; p-value
Tuberculosis				
*Total new enrollments in period range*	129	84	429	
*% treatment coverage*	N/A	7.7	43.2	χ^2^ = 302.45; p < 0.001
*% treatment coverage in PIH-supported districts*	N/A	12.0	67.8	χ^2^ = 412.69; p < 0.001
*Treatment success*	78 (60.5)	58 (69.0)	354 (82.5)	χ^2^ = 8.06; p = 0.005
*Treatment failure*	0 (0.0)	0 (0.0)	2 (0.5)	χ^2^ = 0.39; p > 0.99
*Transfer out*	9 (7.0)	8 (9.5)	34 (7.9)	χ^2^ = 0.24; p = 0.66
*LTFU*	33 (25.6)	8 (9.5)	9 (2.1)	χ^2^ = 12.09; p = 0.003
*Died*	9 (7.0)	10 (11.9)	30 (7.0)	χ^2^ = 2.36; p = 0.13
	Pre-EVD (Jan 2013–Dec 2013)	Pre-intervention (Jan 2015–Jun 2015); n (%)	Post-intervention (Jul 2015–Sep 2016); n (%)	Chi-square statistic; p-value
Leprosy				
*Total new enrollments in period range*	N/A	5	70	
*% treatment coverage*	N/A	16.5	27.6	χ^2^ = 2.92; p = 0.09
*% treatment coverage in PIH-supported districts*	N/A	26.0	43.3	χ^2^ = 3.89; p < 0.05
*Treatment success*	N/A	N/A	59 (84.3)	N/A
*Transfer out*	N/A	N/A	4 (5.7)	N/A
*LTFU*	N/A	N/A	5 (7.1)	N/A
*Died*	N/A	N/A	2 (2.9)	N/A

Note: For pre-post intervention comparisons with fewer than n = 10 observed outcomes, Fisher’s exact statistic was used to calculate p-values, in lieu of chi-square statistics. ‘N/A’ is used to indicate that reliable treatment outcome data was not available.

Primary indicators collected for TB, HIV and leprosy outcomes include ‘total enrollment’; ‘treatment coverage’, or the proportion of expected patients enrolled in care, using appropriate incidence rates; ‘transfer out’ where the patient transferred to another treatment facility; ‘LTFU’; ‘died’; ‘treatment failure’ for those patients completing treatment but testing smear positive; and ‘treatment success.’ For TB patients, treatment success aggregately refers to both patients confirmed cured with smear microscopy and those confirmed clinically who had their treatment stopped. All leprosy treatment outcomes were confirmed clinically as testing was not available during the observed period, thus treatment failure outcomes were not possible to report. Pre-intervention leprosy data at both the county and nation-level were also found to be largely unreliable or incomplete. All deaths and transfers out were confirmed by the Clinical Supervisor of the facility’s ART or chest clinic; if a patient went missing from care and treatment for an unconfirmed reason, they were classified as LTFU. Viral load and CD4 count for HIV patients were not available prior to, or during, the intervention period. All patient outcome information is found in a column of the ART and chest clinic registers.

We disaggregated treatment outcomes for HIV, TB and leprosy patient cohorts by month registered in the respective periods: January 2015–September 2016 for both TB and leprosy, and January 2015–July 2017 for HIV. Treatment coverage for pre- and post-intervention was calculated at the end of June 2015 and the end of June 2017, respectively. Baseline coverage was calculated using estimated disease populations for both Maryland County and national prevalence rates. Coverage post-intervention included estimates of new patients infected since July 2015, using national incidence rates for TB and leprosy; HIV population figures were calculated using Maryland County’s reported incidence rate of 1.5% [5].

Leprosy enrollment and treatment outcome data were not available for Maryland County prior to 2015, as the registers were either extensively damaged by water or vermin, or were lost when one of the facilities moved locations. The MOH did not begin entering leprosy reports into the DHIS-2 database until 2017 and thus patient data was only available through Clinical Supervisor Reports collected by PIH data clerks. For this reason, statistical analysis of leprosy treatment outcomes have been excluded from this manuscript as both pre-intervention and pre-EVD figures are unavailable.

### Statistical analysis

We tested for statistical significance by fitting logistic regression analysis to compare differences in binary outcomes where time was the predictor, using the Pearson’s chi-squared test for direct comparison of the two proportions. For pre-post intervention comparisons with fewer than n = 10 observed outcomes, Fisher’s exact statistic was used to calculate p-values, in lieu of chi-square statistics. A two-sided p-value < 0.05 was considered significant. Statistical analyses were performed using Stata 14.0 SE.

## Results

**Figure 3.**
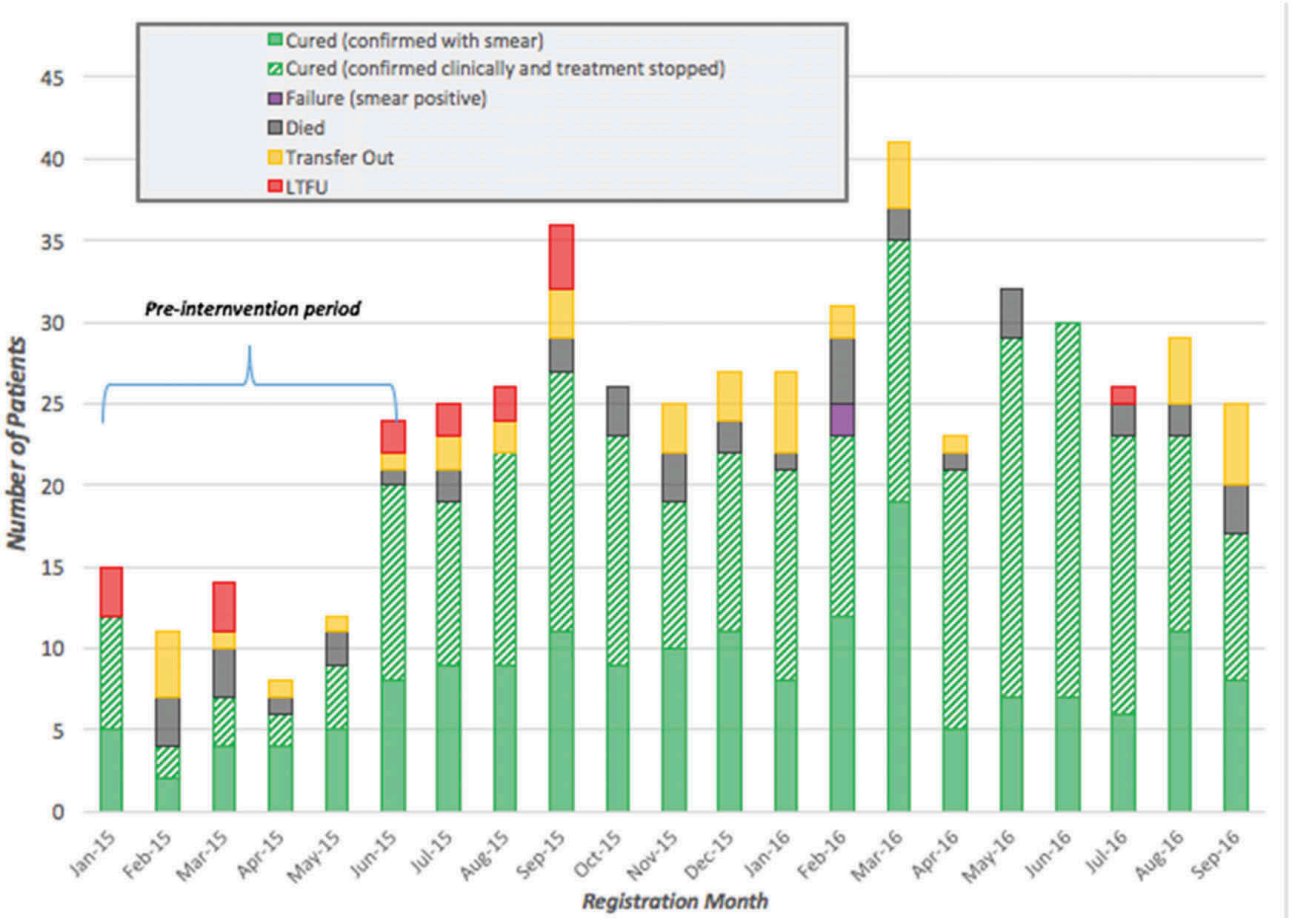
Tuberculosis treatment outcomes for 513 patients across all PIH-supported facilities enrolled between January 2015 and September 2016, disaggregated by month registered, Maryland County, Liberia.

**Figure 4.**
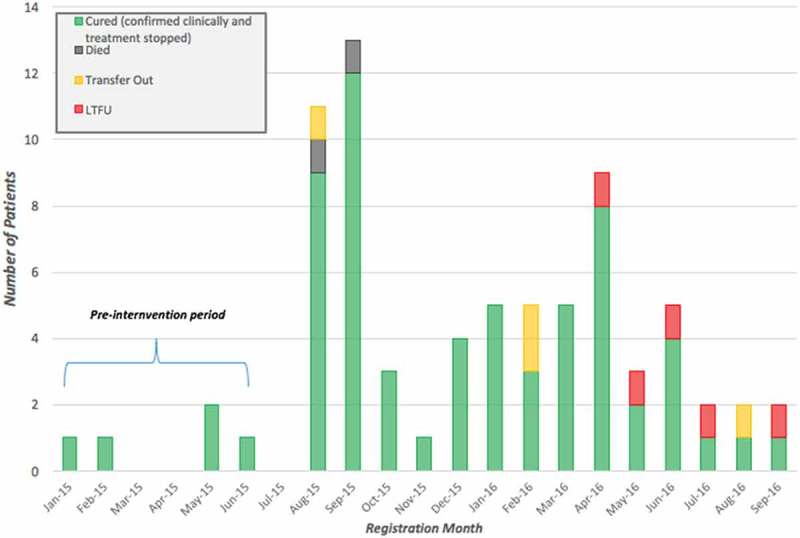
Leprosy treatment outcomes for 75 patients across all PIH-supported facilities enrolled between January 2015 and September 2016, disaggregated by month registered, Maryland County, Liberia.

**Figure 5.**
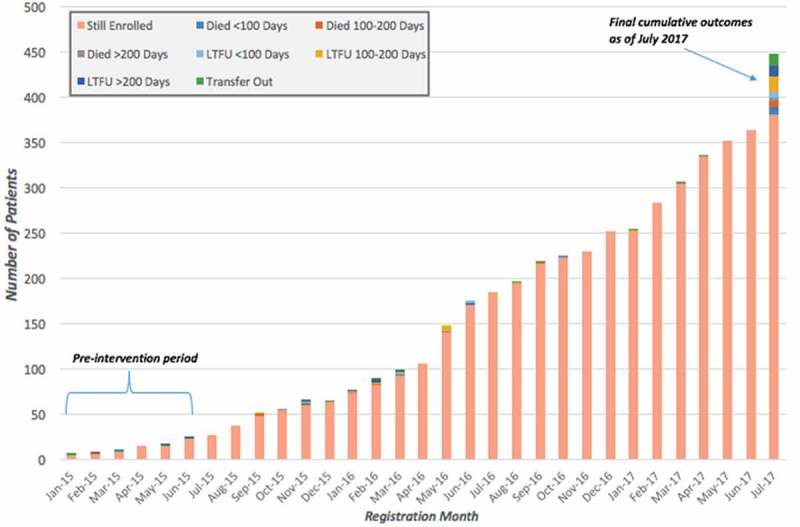
HIV treatment outcomes for 447 patients across all PIH-supported facilities enrolled between January 2015 and July 2017, disaggregated by month registered, Maryland County, Liberia.

We disaggregated treatment outcomes for HIV, TB and leprosy patient cohorts by month registered in the respective periods: January 2015–September 2016 for both TB and leprosy, and January 2015–July 2017 for HIV. We compared the outcomes of 36 HIV, 84 TB, and five leprosy patients enrolled in the pre-intervention period (January 2015 to June 2015) and 411 HIV, 429 TB, and 70 leprosy patients enrolled in the post-intervention period (July 2015 and after). These data are shown in Table 1 and Figures 3–5. Final outcomes of those enrolled during the observed periods were assessed as of July 2017. We observed an increase in new enrollments for HIV, TB and leprosy (1,042%, 411%, and 1,400% respectively) throughout the intervention period.

Several changes in HIV treatment coverage and outcomes observed are significant: retention of HIV patient enrollment increased from 6.9% to 86.1% (χ^2^ = 12.40; p < 0.001), and transfer out dropped from 16.7% to 1.9% (χ^2^ = 23.64; p < 0.001). Change in treatment coverage was found to be of greater significance in PIH-supported districts, increasing from 48.0% to 53.9% (χ^2^ = 6.96; p = 0.008) as compared to treatment coverage throughout Maryland County found to be 30.6% pre-intervention and 34.4% post-intervention (χ^2^ = 4.87; p = 0.03). A marginal decrease in LTFU, from 13.9% to 7.8%, was observed as well as the death rate pre-post intervention, which decreased from 5.6% to 4.1%. Significant improvements in treatment outcomes are observed when post-intervention data is compared to the patient outcomes reported in DHIS-2 for Maryland pre-EVD (Table 1): retention in care improved 52.8 percentage points; the death rate decreased 13.1 percentage points; LTFU decreased 28.3 percentage points; and transfers out decreased 11.4 percentage points. Figure 5 shows a continued increase of new patient enrollments post-intervention, however average monthly enrollment widely varied: the highest being 42 new enrollments recorded in May 2016, the lowest number of new enrollments being three recorded in January 2017.

Improved TB outcomes observed between the pre- and post-intervention periods are also significant. The LTFU rate dropped from 9.5% to 2.1% (χ^2^ = 12.09; p = 0.003), and TB patients enrolled post-intervention had an 82.5% treatment success rate when compared to a baseline 69% success rate (χ^2^ = 8.06; p = 0.005). Associations between the intervention and a reduced death rate did not reach significance, with a 4.9 percentage point decrease (χ^2^ = 2.36; p = 0.13). Changes observed for transfers out (−1.6 percentage points) and treatment failure (0.5 percentage points) were not found to be statistically significant. Treatment coverage throughout Maryland County increased from 7.7% pre-intervention to 43.2% post-intervention (χ^2^ = 302.45; p < 0.001), while in PIH-supported districts treatment coverage increased 55.8 percentage points (χ^2^ = 412.69; p < 0.001). Case detection peaked in March 2016 with 47 new patients enrolled in TB treatment, though new enrollments were consistently higher than the preceding pre-intervention months throughout the duration of the observed intervention period. Out of the 460 patients enrolled on treatment between October 2015 and September 2016, only one was recorded as LTFU, or 0.2% of enrollments. Monthly recorded deaths remained relatively consistent throughout the post-intervention period.

Due to the incomplete availability of pre-EVD or pre-intervention data for leprosy patients, we were not able to determine statistical significance of improved treatment outcomes observed from baseline, and were thereby excluded from Table 1 and subsequent analysis. Leprosy treatment coverage increased by 22.8 percentage points in districts supported by PIH’s primary health care interventions, when compared to an 11.1 percentage point increase throughout Maryland County at large. Post-intervention, we observed a treatment success rate of 84.3% and an LTFU rate of 7.1%, the latter of which is noteworthy when compared to the national leprosy LTFU rate of 64% captured in 2011 [24]. There is a notably consistent trend of leprosy LTFUs for those enrolled between April and September 2016, whereas zero LTFU were reported for those enrolled in all preceding months of the post-intervention period.

## Discussion

Our results show how the introduction of community-based treatment support for HIV, TB and leprosy management in Southeastern Liberia is associated with improved LTFU rates and treatment success for TB, and increased retention and decreased transfers out for HIV clients. The results of our study are noteworthy when compared to the 77% national TB treatment success rate observed in 2016, and the 70% national retention rate at 12 months after initiation on ART reported that same year [17,20]. In the context of our intervention districts, this progress serves as a promising example that despite a large-scale public health emergency such as the West African EVD outbreak, excellent outcomes can be achieved by CHW programs if they are empowered by a functioning health system and additional social supports. While LTFU rates for leprosy were not statistically significant, the post-intervention rate is notably 56.9 percentage points lower than the national average, as noted in the results section [24]. Further study is needed to determine the relationship between death rates and treatment failure for all three targeted diseases and the interventions studied.

While proportional coverage for expected HIV patients was not found to be statistically significant across Maryland County, the increased coverage in PIH-supported districts is noteworthy when compared to national coverage estimated at 19% in 2016 [20]. These results suggest that case finding activities conducted by CHWs in the community – through door-to-door awareness, community outreach, and radio health education broadcasts – improve case identification through facility referrals and follow-up. The increase in new enrollments for HIV, TB and leprosy throughout the 24-month intervention period represents the overall heightened demand for facility-based health care and the improved services being offered. While we acknowledge that there are limitations to the shorter pre-intervention period observed in determining the causal relationship between CHWs and increased enrollment, pre-EVD enrollment figures help to support our argument that increased enrollment in the post-intervention period is not solely a reactionary uptake in services following the epidemic. Pre-EVD, there were 129 new TB enrollments within a 12-month period; comparatively, the first 12 months of our intervention coincided with 349 new TB enrollments, a 170% increase in case detection. Our post-intervention treatment coverage in both PIH-supported districts and Maryland County at-large exceed successful outcomes of CHW impact on case detection reported in Bangladesh, where 50% treatment coverage was estimated. While all HIV treatment outcomes post-intervention are notably improved from pre-EVD outcomes, enrollment trends observed in the post-intervention period closely reflect pre-EVD figures, indicating that the sharp increase between pre- and post-intervention is a reaction to health facilities returning to their prior states of functionality. Further study is required to determine why the impact of the CHW intervention on HIV case detection was less significant than for TB, though conjectures related to the absence of food support for ART patients as incentive for testing and enrollment, as well as the prodigious levels of stigma and discrimination surrounding the disease within Liberian communities, can be made.

Improved retention in care for ART patients further support that a driver of change in this intervention was the provision of high quality care that simultaneously addressed the target population’s vulnerability with material investments – especially when compared to the reported rate of 33.3% in Maryland County pre-EVD. We define high quality care as access to a facility with trained professional staff, an unbroken supply chain, and access to reliable diagnostics as needed. We aimed to address vulnerability by two primary mechanisms: first, employing CHWs to perform community-based active case finding and then extending CHW-mediated treatment support that offered educational and psychosocial support tailored to the patients’ needs; second, offering patients material supports that offered the tools necessary to be adherent. More specifically, these supports consist of food to lessen the clinical dangers of a catabolic state and to support the patients through the pains of taking oral medicines daily, and transport reimbursement to facilitate clinic follow-up. These are offered because of the observation that despite many patients’ best intentions, treatment failures are a product of limited agency borne from extreme poverty; material supports augment the patients’ ability to be an active agent in improving treatment outcomes. This approach has resulted in treatment successes across many different contexts and a variety of disease burdens – in Rwanda, the provision of transport reimbursement, food support packages, and social support resulted in 92.3% of ART patients retained in care [26], and in Afghanistan a qualitative evaluation of a nutritional support program for TB suggested food is an effective adjunctive to enhance adherence [27].

Our program was able to achieve TB treatment success rates comparable to those achieved by other studies introducing community-administered DOTS, such as the intervention in Bangladesh that observed a cure rate of 85% and one in post-conflict Afghanistan that observed an 86% treatment success rate. The intervention in Afghanistan also involved concurrent, rapid expansion of the number of facilities capable of providing TB diagnostic and treatment services. Throughout our intervention, CHWs did not directly provide DOTS due to high patient loads and wide geographic displacement of enrolled patients. Instead, the program focused on providing psychosocial support, continuous health education and delivery of the socioeconomic assistance to patients. Our outcomes obfuscate findings from studies that have heralded community-administered DOTS to be a paramount component of TB treatment and improved outcomes [15]. Outcomes of our intervention are impressive despite the limitations of our CHW cohort’s size and geographical reach to provide routine home visits, and a lack of decentralized clinical care for ART, TB and leprosy patients in four out of the six health districts in Maryland County .

It bears mentioning that investments in adherence support like food and transport are rarely included in national budgets as an essential component of treatment programs for chronic diseases, yet our practical experience suggests that they are indispensable for achieving excellent clinical outcomes. This is especially true in impoverished and fragile countries, but current funding mechanisms make it difficult to implement these types of programs at scale. We recommend that global public health practitioners and researchers reconsider the assumptions that generate such underperformance. More research is needed to assess the return on investment of such socioeconomic interventions and the costs associated with LTFU reduction.

Researchers and implementers seeking to accomplish similar successes with CHW programs should also provide concurrent investment in decentralized clinical support that ensures consistent availability of trained staff, test kits, and medication at health facilities to which CHWs are referring patients. It is recommended that increased investment in such inputs, by implementing partners and policymakers alike, be made in post-emergency contexts to strengthen gaps in rural health care delivery.

### Limitations

Our analyses are confounded by the end of the EVD outbreak, which coincided with our observed pre-intervention period, and could account for some decreases in LTFU rates and naturally increased coverage rates. A truncated pre-intervention period was observed when compared to our post-intervention period due to monthly-reported longitudinal data not being available prior to 2015, acting as another limitation to the study’s results. In the absence of a control group, we cannot say with certainty that the outcomes observed were caused by any specific component of the intervention, but the dramatic change makes a strong case that this approach is capable of generating similar outcomes in other destabilized contexts. Furthermore, we cannot ascertain with any level of certainty the impact that the CHWs’ educational background has had on the success of the intervention as literacy levels of the CHWs were not evaluated during the recruitment process. More research is needed to better understand the relative contributions of each intervention, and to further delineate the direction and causality of associations that remain unclear. In the context of a recent emergency, we did not have the time necessary to implement an experimental design, but future interventions can be designed with cutting-edge implementation science techniques such as stepped-wedge trials.

## Conclusion

To summarize, our study shows that a comprehensive and integrated CHW program that provides social supports for patients can result in increased case finding, improved treatment completion, and decreased LTFU outcomes for TB and HIV patients. Improved retention and decreased transfers out for HIV patients was also observed, while its impact on leprosy treatment outcomes was inconclusive. We recommend increased investments in both food support and transportation reimbursement for chronic infectious disease patients be made to facilitate access to care and treatment adherence. We suggest that additional research and cost benefit analysis of this socioeconomic assistance model be conducted to ascertain whether sustainable scale-up, at a national or regional level, is feasible.

## Paper context

Evidence assessing multi-faceted interventions on treatment outcomes for chronic infectious disease patients, specifically ones addressing the intervention population’s socioeconomic vulnerabilities, is lacking. This study found that integration of community, facility and social support through CHW programs correlate with improved clinical outcomes, even in post-emergency settings. Recommendations can be made for adopting an integrated community treatment support model as this approach may improve long-term retention in care and help bridge the rural health delivery gap.
